# Relationship and Effects of Vitamin D on Metabolic Syndrome: A Systematic Review

**DOI:** 10.7759/cureus.17419

**Published:** 2021-08-24

**Authors:** Nyein Wint Yee Theik, Oluwatimilehin E Raji, Priya Shenwai, Rutul Shah, Sahithi Reddy Kalluri, Tinaz H Bhutta, Hanan Hannoodee, Mahmoud Al Khalili, Safeera Khan

**Affiliations:** 1 Internal Medicine, California Institute of Behavioral Neurosciences & Psychology (CIBNP), Fairfield, USA

**Keywords:** low vitamin d, rickets, metabolic syndrome, obesity, glucose insensitivity, insulin resistance, dyslipidemia, high blood pressure

## Abstract

Metabolic syndrome (MetS) is a persistent public health problem in the United States (U.S.) due to its increasing prevalence and its positive correlation with type-2 diabetes (T2DM) and cardiovascular disease (CVD). According to National Cholesterol Education Program's Adult Treatment Panel III (NCEP-ATP III) criteria, MetS has six main components, which are obesity, dyslipidemia, raised blood pressure (BP), insulin resistance (IR) or glucose intolerance, pro-inflammatory state, and prothrombotic state. Vitamin D (Vit D) regulates the absorption of calcium and phosphorus and thus, is universally accepted as an essential vitamin for bone strength as well as a facilitator of immune system function. Vit D was also shown to reduce the risks of CVD, multiple sclerosis, and developing seasonal flu.

We conducted a systematic review to identify the general association between Vit D level and MetS, to highlight specific associations between Vit D level and individual components of MetS, and finally, to explore the effects of Vit D supplementation on each component of MetS.

In this paper, we reviewed 14 recent studies investigating the relationships between Vit D, MetS, and components of MetS. From the review of seven studies, we confirmed a significant association between Vit D and MetS as a whole. Four out of the five observational studies we reviewed support that Vit D level is significantly associated with the following components of MetS: obesity and BMI, dyslipidemia, BP, and insulin and glucose metabolism.

We did not discover any significant relationship between Vit D level and other MetS components. The review of seven additional randomized clinical trials (RCT)-based studies suggest that Vit D supplementation has significant effects on BP, abdominal obesity, and insulin and glucose metabolism.

## Introduction and background

Metabolic syndrome (MetS) in the United States (U.S.) adults 18 years or older increased from 25% in 1988 to 34% in 2012 [1]. A more recent study by Hirode and Wong found that the prevalence of MetS among the U.S. population increased slightly to 36.9% as of 2016 [2]. MetS is known as one of the most important risk factors of type-2 diabetes (T2DM) and cardiovascular disease (CVD), which in turn can increase the risk of myocardial infarction and stroke two-fold [3,4]. According to the Centers for Disease Control and Prevention (CDC), patients with some form of MetS are also likely to be more affected by COVID-19 [5].

According to the National Cholesterol Education Program's Adult Treatment Panel III report (NCEP-ATP III), there are six components of MetS related to CVD. They are abdominal obesity, atherogenic dyslipidemia, raised blood pressure (BP), insulin resistance (IR) or glucose intolerance, pro-inflammatory state, and prothrombotic state [6,7]. Despite being known as a serious public health issue, MetS does not have any straightforward, definitive treatment due to its multifaceted nature [8]. The most common clinical management of MetS is to reduce the risk factors; for example, maintaining low-density lipoprotein (LDL) cholesterol levels and BP under a certain threshold, mitigating the risk of T2DM for those who belong to at-risk populations, and encouraging patients to adopt lifestyle changes including eating a healthy diet and doing regular exercise [8,9].

Vitamin D (Vit D) is a fat-soluble, essential vitamin that facilitates calcium absorption. Vit D is produced in the human body when the skin is exposed to ultraviolet rays from sunlight. Vit D is also available from certain foods such as oily fish, egg yolks, red meat, liver, and in certain vitamin supplements [10]. Inadequate Vit D level commonly leads to osteomalacia in adults and rickets in infants [11]. The daily dietary allowance of Vit D is 400 - 800 IU in humans depending on age and sex [12]. However, physicians sometimes prescribe more than 4000 IU to compensate for the Vit D deficiency [13]. 

Vitamin D deficiency is generally accepted as below 50ng/mL of 25-Hydroxyvitamin D concentration in blood [11]. Forrest and Stuhldreher found that Vit D deficiency is 41.6% in the general U.S. population but can be as high as 82.1% among African Americans and 69.2% among Hispanics [14].

Researchers have conducted several studies to establish the relationship between Vit D and specific components of MetS. For example, some studies investigated the effects of Vit D on BP, triglycerides (TG), and glucose intolerance [15-21]. Some studies explore the association between Vit D and MetS as a whole [22, 23]. Some of the studies deployed randomized clinical trials (RCTs), whereas some relied on observational studies. The population recruited for these studies vary by sex, age, and country of origin [19,23-25]. A few studies reported a non-significant association between Vit D and different components of MetS [24-26]. By organizing these disparate studies into one systematic review, we hope to highlight the commonalities and differences in these studies, providing a big-picture analysis and understanding of the correlation between Vit D and MetS that applies to the general adult population around the world. This systematic review aims to illustrate the relationship between Vit D level and different components of MetS and the effects of Vit D supplementation on components of MetS. Based on the findings in this systematic review, we hope to accomplish two goals in our future studies: first, to identify the threshold of Vit D levels that can be used as a risk indicator of MetS for specific demographics, and second, to identify the specific dosage of Vit D that can reduce the risk of CVD.

## Review

Method and results

We used the Preferred Reporting Items for Systematic Reviews and Meta-Analyses (PRISMA) guidelines and principles in designing this systematic review and reporting its results [27].

Search strategy

Using the major research literature database and search engines such as PubMed, PubMed Central (MEDLINE), Google Scholar and, ResearchGate, we searched for appropriate keywords and medical subject headings (MeSH) thesaurus to uncover potentially relevant articles demonstrating the relationship between Vit D and MetS [28]. The keywords used in our literature search include "low vitamin D", "hypovitaminosis D", "rickets", "metabolic syndrome", "obesity", "glucose insensitivity", "insulin resistance", "dyslipidemia" and "high blood pressure". We searched for their corresponding MeSH terms using PubMed Central [29]. The combined MeSH terms for all of the aforementioned keywords are as follows: low vitamin D OR hypovitaminosis D OR rickets OR ("rickets/complications" [MeSH] OR "rickets/diet therapy" [MeSH] OR "rickets/drug therapy" [MeSH] OR "rickets/therapeutic use" [MeSH] OR "rickets/therapy" [MeSH] ) AND metabolic syndrome OR obesity OR glucose insensitivity OR insulin resistance OR dyslipidemia OR high blood pressure OR ("metabolic syndrome/diet therapy" [MeSH] OR "metabolic syndrome/drug therapy" [MeSH] OR "metabolic syndrome/prevention and control" [MeSH]). We also searched for the full text of the relevant journal articles using Google Scholar and ResearchGate [30,31].

Inclusion and exclusion criteria

We selected the studies published within the past five years (2017 to 2021) in English. We filtered for systematic reviews, analytical studies, experimental studies with RCTs, and observational studies. In addition, we only selected studies with human subjects. We ensured that most patients recruited in these studies were between the ages of 13 and 75 years.

Analysis of study quality

The RCTs were critically evaluated with the Cochrane bias assessment tool, systematic reviews were assessed using the assessment of the multiple systematic reviews (AMSTAR) tool, and observational studies were analyzed with the Newcastle-Ottawa assessment scale (NOS). Each research work was scored as either a high, medium, low or unclear quality, and we selected the ones with medium and high-quality scores in our final analysis. The overall quality and scores for each study are provided in Table 1, Table 2, and Table 3.

**Table 1:** A tabulated summary of the Cochrane risk of bias tool

Cochrane criteria (Yes, No, Uncertain)	Cefalo et al. 2018 [20]	Lerchbaum et al. 2019 [19]	Safapour et al. 2020 [21]	Raed et al. 2017 [15]	McMullan et al. 2017 [16]	Karefykalis et al. 2018 [26]	Angellotti et al. 2019 [24]
Adequate sequence generation?	Yes	Yes	Yes	Yes	Yes	Yes	Yes
Allocation concealment used?	Uncertain	Uncertain	Uncertain	Uncertain	Uncertain	Uncertain	Uncertain
Blinding?	Yes	Yes	Yes	Yes	Yes	Yes	Yes
Are concurrent therapies similar?	Yes	Yes	Yes	Yes	Yes	Yes	Yes
Incomplete outcome data addressed?	Yes	Yes	Yes	Yes	Yes	Yes	Yes
Uniform and explicit outcome definitions?	Yes	Yes	Uncertain	Yes	Yes	Uncertain	Yes
Free of selective outcome reporting?	Yes	Yes	Yes	Yes	Yes	Yes	Yes
Free of other bias?	Yes	Yes	Yes	Yes	Yes	Yes	Yes
Overall risk of bias?	Yes	Yes	Yes	Yes	Yes	Yes	Yes
Our Evaluation	8/9 (High quality)	8/9 (High quality)	7/9 Medium quality)	8/9 (High quality)	8/9 (High quality)	7/9 (Medium quality)	8/9 (High quality)

**Table 2:** A tabulated summary of the Newcastle-Ottawa risk of bias tool

Newcastle-Ottawa criteria (Yes, No, Uncertain)	Khoja et al. 2017 [17]	Mirhoseini et al. 2018 [18]	Krishnaswamy et al. 2019 [23]	Shamy et al. 2020 [32]	Kaminska et al. 2020 [25]	Fite et al. 2020 [33]
Representative of the exposed cohort	Uncertain	Uncertain	Uncertain	Uncertain	Uncertain	Uncertain
Selection of external control	Uncertain	Uncertain	Uncertain	Uncertain	Uncertain	Uncertain
Ascertainment of exposure	Yes	Yes	Yes	Yes	Yes	Yes
Outcome of interest not present at the start of the study	Yes	Yes	Yes	Yes	Yes	Yes
Comparability of main factor	Yes	Yes	Yes	Yes	Yes	Yes
Comparability of additional factor	Uncertain	Uncertain	Uncertain	Uncertain	Yes	Uncertain
Assessment of outcome	Yes	Yes	Yes	Yes	Yes	Yes
Sufficient follow-up time	Yes	Yes	Yes	Yes	Yes	Yes
Adequacy of follow-up	Yes	Yes	Yes	Yes	Yes	Yes
Our evaluation	6/9 (Medium quality)	6/9 (Medium quality)	6/9 (Medium quality)	6/9 (Medium quality)	7/9 (Medium quality)	6/9 (Medium quality)

**Table 3:** A tabulated summary of the AMSTAR risk of bias tool

AMSTAR criteria (Yes, No, Uncertain)	A priori design	Duplicate study selection and data extraction	Literature search	Status of publication	List of studies	Characteristics of included studies	Scientific quality	Formulation of conclusion	Method of used to combine findings	Likelihood of publication bias	Conflict of interest	Our evaluation
Hajhashemy et al. 2021 [22]	Uncertain	Uncertain	Yes	Yes	Yes	Yes	Uncertain	Yes	Yes	Yes	Uncertain	7/11 (Medium quality)

Results

**Figure 1:**
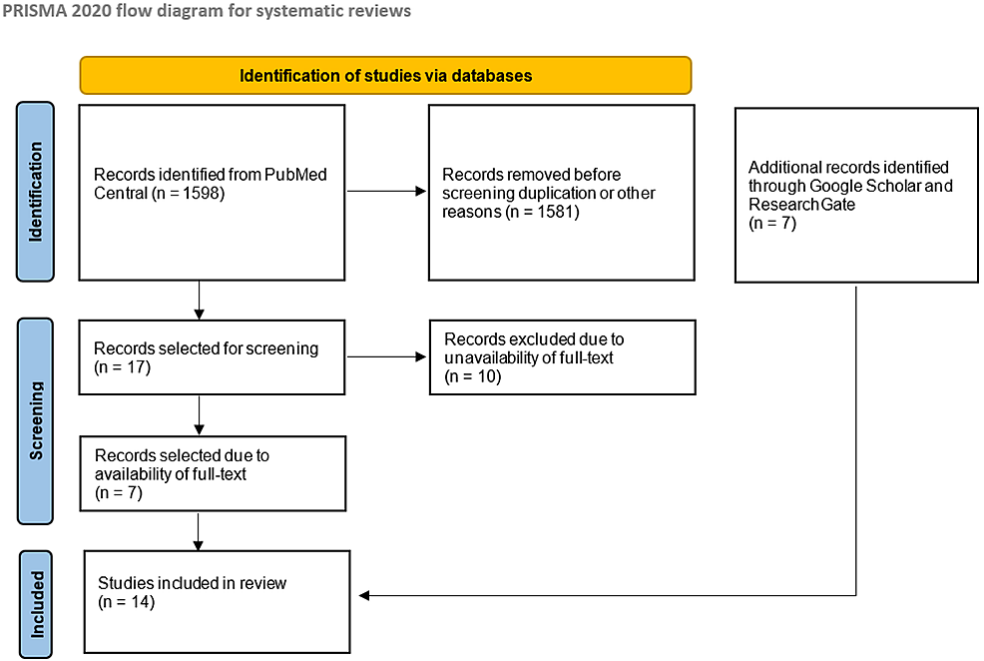
Preferred Reporting Items for Systematic Reviews and Meta-Analyses (PRISMA) flow diagram

A total of 1598 articles were identified in our initial search of the PubMed Central (MEDLINE) database. Out of these articles, 1581 were discarded either due to duplication or not being directly relevant to our research focus, leaving us with 17 articles to analyze. In these 17 research papers, we were able to access the full text of seven papers; the other 10 could only be obtained via paid subscription, and thus, we excluded them. To compensate for the discarded articles, we used Google Scholar and ResearchGate to obtain seven more articles that met our inclusion and exclusion criteria. A total of 14 articles were reviewed in this paper. We provide the complete PRISMA flow diagram in Figure 1 below.

Discussion

A 2002 study by Ogunkalade et al. is the earliest work that proved the association between Vit D receptor polymorphism and insulin secretory capacity [34]. Malin et al. in 2014 showed that insulin secretory capacity and beta-cell dysfunction are the most prominent factors that are correlated with the severity of MetS [35]. Following these findings, several more papers revealed the association between Vit D and MetS and the effects of the former on the latter. Among them, we selected 14 papers published since 2017, which summarize the relationship between Vit D level and MetS. In addition, these papers reported the effects, or lack thereof, of Vit D level on each of the six major components of MetS. To be generalizable for most adult populations, we selected different research types consisting of experimental and observational studies, a systematic review, and an analytical study.

Relationship of Vit D with each of the six major components of MetS

From 2017 to 2020, we selected four observational studies investigating the association between Vit D and the components of MetS. These studies have the same objective i.e., to investigate the association between Vit D and MetS. However, they differ in sample size, locale, and the demographic of the trial participants, as well as case-control criteria.

In a study by Mirhoseini et al., 192 obese patients with body mass index (BMI) > 25 from two hospitals in Iran's third-largest city were enrolled [18]. Patients were divided into healthy obese, which means obese without MetS, and obese with MetS. NCEP-ATP III criteria of TG level > 150mg/dL, high-density lipoprotein C (HDL-C) < 40mg/dL in men and < 50mg/dL in women, and fasting blood sugar (FBS) > 100mg/dL were applied in determining the participants of MetS group [6,36]. After 12 hours of fasting, Vit D level was measured for each group. Based on the laboratory report, a Vit D level of < 20ng/mL is defined as low, a level of 20-30ng/mL as insufficient, and > 100ng/mL is defined as toxic. The components of MetS in focus for this study were TG, HDL-C, FBS, BP, waist circumference (WC), and BMI. Among these components, TG, HDL-C, FBS, and BP were significantly associated with Vit D levels. As mentioned, Vit D is a fat-soluble vitamin. Although it was shown that Vit D levels become more diluted as lipid volume increases, the study did not find any significant association between Vit D and obesity-related indicators such as WC and BMI [37].

In a cross-sectional study by Krishnaswamy et al., 80 Indian patients aged between 18 and 60 years were divided into MetS (case) and healthy (control) groups [23]. The Joint Interim Statement (JIS), which was issued by world-renowned organizations such as the International Diabetes Federation and the American Heart Association, was applied when selecting patients in the MetS group [38]. The study found that Vit D level in the case group was significantly lower than that in the control group. The relationships between Vit D and each component of MetS, however, were not reported in detail. Although the study reported that low Vit D level is significantly associated with MetS, the overall Vit D levels in both case and control groups were lower than the normal range (20 to 40ng/mL). This could be explained by the fact that Vit D deficiency (VDD) is prevalent in India [39]. The study was also limited not just by its small sample size but by its participants only being recruited from the hospital's outpatient department.

In a case-control study by Shamy et al., 92 patients from an Egyptian hospital's outpatient department, who are older than 18 years, were divided into MetS (case) and healthy (control) groups [32]. JIS criteria were used to determine the patients in the MetS group. The parameters of MetS in focus for this study were TG, high-density lipoprotein (HDL), lipid accumulation product (LAP), FBS, systolic blood pressure (SBP), diastolic blood pressure (DBP), BMI, WC, alanine aminotransferase (ALT), and aspartate aminotransferase (AST). Contrary to the studies discussed above, no significant difference in Vit D level between the case and control groups was found. In particular, the researchers found no significant correlation between Vit D vs. TG, Vit D vs. FBS, Vit D vs. SBP, Vit D vs. DBP, Vit D vs. BMI, and Vit D vs. WC. The only two significant findings from this study were the inverse associations between Vit D vs. HDL and Vit D vs. LAP. In this study, LAP was calculated as follows: LAP for men = (WC in cm - 65) x TG (mmol/L) and LAP for women = (WC in cm - 58) x TG (mmol/L) [40]. The findings indicate that although WC and TG were used to calculate LAP, which was found to be significantly associated with Vit D level, neither WC nor TG was individually associated with Vit D. Similar to the study by Krishnaswany et al., this study recruited hospital outpatients as participants. However, most of the components of MetS, except LAP and HDL, were not significantly associated with Vit D level. The contradictory findings from these two studies could be due to the difference in the inherent prevalence of VDD in Egypt vs. India and other compounding factors such as lifestyle differences between the two countries.

A cross-sectional study by Khoja et al. investigated the association between Vit D receptor (VDR) genes and MetS and the association of Vit D and each component of MetS [17]. Researchers focused on four variables of VDR gene polymorphism - namely ApaI (VDR 7975232 C > T), BsmI (VDR 1544410 A > G), FokI (VDR 2228570 C > T), and TaqI (VDR 731236 T > C) - and reported that MetS is significantly associated with ApaI gene polymorphism. In this study, 82 patients from Saudi Arabia between the age of 20 and 50 years were divided into MetS (case) and healthy (control) groups. The patients in the case group have three out of five NCEP-ATP III criteria. The researchers found that patients in the MetS group have significantly low Vit D levels than those in the control group. Among the six components of MetS, Vit D is significantly and inversely associated with each of the following factors: TG, hemoglobin A1C (HbA1C), BP, and WC. Unlike other studies discussed thus far, we should note that this study measured HbA1C instead of FBS for IR or glucose intolerance.

An observational study by Kaminska et al. recruited 2381 Polish patients. Similar to the study by Krishnaswamy et al., patients for this MetS (case) group were selected using the JIS criteria [25]. The patients were subdivided into male and female. Researchers found a significant inverse relationship between Vit D level and SBP among the female population and no significant finding among the male population. Male and female participants were then further subdivided into two groups using a BMI threshold of 30. A significant association between Vit D vs. TG and Vit D vs. BP was found for BMI > 30. Similarly, for men with BMI > 30, there is a significant association between Vit D vs. HDL and between Vit D vs. BP. For men with BMI < 30, Vit D level is significantly associated with WC. Although only one of the MetS components, SBP, was significantly associated with Vit D for female participants, more significant associations were identified after further dividing the participants by BMI. Similar to the study by Mirhoseini et al., this study found no significant association between Vit D and WC for men who are above the BMI threshold. Compared to other studies discussed before, this study has the largest participant population and was able to detect the significant association between Vit D and some components of MetS for specific gender and BMI levels.

Although each of the studies above observed significant relationships between Vit D and some components of MetS, none of them observed a significant association between Vit D and all of the components of MetS. The studies differ greatly in the number of participants and their demographics, as well as the study types. Thus, we must be careful not to draw generalizable conclusions regarding the association between Vit D and all MetS components. In other words, differences in participant demographics, varying size of participant groups, and other disparities such as diet and the climate of each population could have significantly affected the outcomes and findings of these studies. In our quest to find a more generalizable finding, we examined a systematic review and a paper that analyzed the data from the 2005-2006 National Health and Nutrition Examination Survey (NHANES). Both studies have large sample sizes covering more than one demographic or locale.

The first of these studies was a systematic review with the meta-analysis by Hajhashemy et al., which reviewed 38 cross-sectional studies, four cohort studies, and one case-control study [22]. A total of 309,206 adult participants were included in this systematic review. The participants represented 24 geographically diverse countries such as the U.S., China, Korea, Thailand, Qatar, Australia, Taiwan, Iran, Northern Finland, India, South Africa, Portugal, Japan, United Kingdom, Germany, Netherland, Italy, Belgium, Poland, Sweden, Spain, Hungary, and Estonia. The studies included in this systematic review used one of the two different criteria -NCEP-ATP III and JIS.

In the systematic review of the 38 cross-sectional studies (N=298,187) by Hajhashemy et al., the researchers found that the highest Vit D level can be translated into 43% and 40% reduction MetS in developed and developing countries, respectively. Due to the sheer number of studies reviewed in the paper and the high heterogeneity of the data, the researchers also conducted a subgroup analysis. The meta-analysis outcome showed a significant relationship between Vit D and MetS for all 38 cross-sectional studies, but not in the other five studies (N=11,019), four cohort studies, and one case-control study. The strength of the systematic review by Hajhashemy et al. is that it analyzed the data from several studies covering the adult population from around the world, which made its findings more generalizable than studies focused on specific locale and demographics.

Fite et al. performed statistical analysis on the 2005-2006 NHANES data [33]. The U.S. non-institutionalized civilian population of 10,348 people was included in this survey. The researchers showed that VDD is significantly associated with MetS across all ages. The study also reported a significant association between VDD and components of MetS, particularly TG, HDL, FBS, IR, BP, and WC. Since the NHANES survey is conducted by highly trained medical personnel and is designed to cover a nationally representative sample of the U.S., a country with diverse racial, cultural, ethnicity and socioeconomic backgrounds, the analysis outcome of such survey data can be very applicable for the general population of a developed nation [41]. However, Fite et al. acknowledged that the sample size in the NHANES survey is not sufficiently large and homogeneous enough to perform stratified analyses based on different population groups.

The brief descriptions of each study, including the year of publication, author name, number of patients and country of origin, type of study, significant and non-significant findings, are provided in Table 4.

**Table 4:** Summary of significant vs. non-significant associations between Vit D and MetS in general, and between Vit D and individual MetS components based on the five observational studies, one analytical study on the NHANES survey data, and one systematic review. Vit D: Vitamin D; MetS: Metabolic syndrome; U.S.: United States; UK: United Kingdom; NHANES: National Health and Nutrition Examination Survey; TG: Triglycerides; HbA1C: Hemoglobin A1C; BP: Blood pressure; WC: Waist circumference; FBS: Fasting blood sugar; BMI: Body mass index; HDL-C: High-density lipoprotein-C; HDL: High-density lipoprotein; LAP: Lipid accumulation product; SBP: Systolic blood pressure; DBP: Diastolic blood pressure; ALT: Alanine aminotransferase; AST: Aspartate aminotransferase; IR: Insulin resistance

Published year	Author	Number of patients and country of origin	Type of study	Overall associations between Vit D and MetS	MetS component(s) significant associations with Vit D	MetS component(s) non-significant associations with Vit D
2017	Khoja et al. [17]	82 (Saudi Arabia)	Observational (cross-sectional)	p < 0.01	TG, HbA1C, BP, WC	FBS, BMI
2018	Mirhoseini et al. [18]	192 (Iran)	Observational (descriptive analytical)	p < 0.001	TG, HDL-C, FBS, BP	WC, BMI
2019	Krishnaswamy et al. [23]	80 (India)	Observational (cross-sectional)	p < 0.05	Not reported	Not reported
2020	Shamy et al. [32]	92 (Egypt)	Observational (case-control)	p < 0.09	HDL, LAP	TG, FBS, SBP, DBP, WC, BMI, ALT, AST
2020	Kaminska et al. [25]	2381 (Poland)	Observational	Not reported	All female: SBP	TG, HDL-C, FBS, DBP, WC
					All male: non-significant	TG, HDL-C, FBS, SBP, DBP, WC
					Female BMI > 30: TG, SBP, DBP	HDL-C, FBS, WC
					Female BMI < 30: non-significant	TG, HDL-C, FBS, SBP, DBP, WC
					Male BMI > 30: HDL-C, SBP, DBP	TG, FBS, WC
					Male BMI < 30: WC	TG, HDL-C, FBS, SBP, DBP
2020	Fite et al. [33]	11,019 (U.S.)	Analytical study on the NHANES survey data	p < 0.001	TG, HDL, FBS, IR., BP, WC	Not reported
2021	Hajhashemy et al. [22]	309,206 (23 countries including U.S., China, India, South Africa, Portugal, UK, Hungary, etc.)	Systematic review and meta-analysis	High Vit D concentration is significantly related to the decrease in the odds of developing MetS	Not reported	Not reported

Effects of Vit D supplementation on components of MetS

Seven out of 14 articles, which we selected for review, used RCTs to detect the effects if any, of Vit D on components of MetS such as BP, insulin, and glucose metabolism. The articles reviewed are different in the length of study, the components of MetS studied, and the findings. In an RCT by Lerchbaum et al., 192 male patients aged between 18 and 70 years with a low Vit D level of < 75nmol/L (< 30ng/mL) were recruited [19]. The RCT was a twelve-week long trial, and the progression of metabolic parameters was regularly monitored while providing Vit D supplements to the participants. In this study, Vit D supplementation was significantly associated with improving fasting glucose to insulin ratio. Other components of MetS, such as dyslipidemia and body composition, were not significantly associated with Vit D supplementation. Since this study was solely conducted on the male population, the significant findings in this study might not be attributable to the female population. In this study, the baseline Vit D level of the selected participants was higher than the commonly-used baseline of 20ng/mL.

In an RCT by Cefalo et al., a significant association between Vit D supplementation on insulin metabolism was observed [20]. RCT by Cefalo et al. lasted 13 weeks and was performed with just 18 patients who had low Vit D < 75nmol/L (< 30ng/mL) and BMI > 25. Insulin sensitivity and total trunk fat mass measured by X-ray absorptiometry were significantly improved with Vit D supplementation. Other components of MetS such as TG, HDL, LDL, and BMI were not significantly associated with Vit D supplementation. 

In an RCT by Safapour et al., 85 patients who have T2DM and BMI > 25 were recruited for the study [21]. To measure insulin and glucose metabolism, researchers used HbA1C, insulin level, FBS, and glucose indices such as sirtuin 1 (SIRT1), irisin, homeostatic model assessment of insulin resistance (HOMA-IR), and quantitative insulin sensitivity check index (QUICKI). Vit D supplementation was found to significantly improve SIRT1 and irisin, which indicates the effect of Vit D on glucose metabolism. Effects of Vit D on other components of MetS were not reported in this study. The findings from the studies by Lerchbaum et al., Cefalo et al., and Safapour et al. agree with Maestro et al.'s study in 2000, which showed that Vit D directly activates the expression of insulin receptors and enhances insulin responsiveness that subsequently improves the metabolism of glucose and insulin [42].

In a 2011 study by Mheid et al., researchers proved the effect of Vit D in suppressing the proliferation of vascular smooth muscle, which in turn affects BP by regulating the renin-angiotensin-aldosterone (RAA) system [43]. Two of the seven RCT-based papers we selected investigated the effects of Vit D supplementation in BP and arterial stiffness. An RCT by Raed et al. recruited 70 African American patients between 13 to 45 years of age who have a Vit D level < 20ng/mL [15]. The trial lasted 18 weeks, and carotid-femoral pulse wave velocity (PWV) and carotid-radial PWV were measured to check the participants' arterial stiffness. The researchers found that Vit D supplementation is significantly associated with a reduction of arterial wall stiffness. 

An RCT conducted by McMullan et al. was an eight-week-long study, and 84 patients with Vit D levels below 20ng/mL were recruited from the greater Boston area [16]. Plasma renin activity (PRA), angiotensin II (Ag II), and 24-hour BP were monitored while the participants were provided with Vit D supplementation. No significant improvement in PRA, Ag II and 24-hour BP was detected with the Vit D supplementation. The studies by McMullan et al. and Raed et al. aimed to discover the effect of Vit D supplementation on BP, and they reached opposing conclusions. Part of such contradictory findings could be explained by the unusually short length of the trial in McMullan et al.'s study. Another possible explanation could be that Raed et al.'s study solely focused on the African American population, who are known to have a higher risk of VDD than the general population, and supplementation of Vit D could be more pronounced and beneficial for them [44].

In an RCT by Karefykalis et al., 40 patients who had BMI > 25 and Vit D < 55nmol/L (< 22 ng/mL) were recruited [26]. The trial was 26 weeks long. The components of MetS studied in it were the percentage of body fat, BMI, maximum oxygen uptake (V02max), and oxygen uptake at anaerobic threshold (AT). V02max and oxygen uptake at AT were measured to investigate the strength of circulation and respiration. No significant changes in these components were detected when the participants were provided with Vit D supplementation. An RCT by Angellotti et al. was performed with 127 participants with HbA1C < 7.5 and lasted for 48 weeks [24]. Like in the trial by Karefykalis et al., Vit D supplementation had no significant effect on the components of MetS studied, including the lipid profile, C-reactive protein (CRP), and CVD risks.

Based on the review of all seven RCTs selected, we found that the researchers demonstrated the effects of Vit D on three main components of MetS, which are BP, abdominal obesity, and insulin and glucose metabolism. The studies did not find Vit D to significantly affect other components of MetS such as dyslipidemia, pro-inflammatory state, and prothrombotic state. A notable limitation of our RCT papers is the small number of participants in each of the trials - six out of seven studies in these papers were performed with less than a hundred participants.

The brief descriptions of each study, including the year of publication, author name, number of patients and country of origin, duration of the study, significant and non-significant findings, are provided in Table 5. 

**Table 5:** Summary of significant vs. non-significant findings on the effects of Vit D supplementation on six major components of MetS based on the seven RCT papers reviewed. MetS: Metabolic syndrome; Vit D: Vitamin D; U.S.: United States; BP: Blood pressure

Published year	Author	Number of patients	Duration of study	MetS component(s) significantly improved with Vit D supplementation	MetS component(s) non-significant associations with Vit D supplementation
2018	Cefalo et al. [20]	18 (Italy)	13 weeks	Insulin and glucose metabolism, and abdominal obesity	Dyslipidemia
2019	Lerchbaum et al. [19]	192 (Austria)	12 weeks	Insulin and glucose metabolism	Dyslipidemia, abdominal obesity
2020	Safapour et al. [21]	85 (Iran)	Eight weeks	Insulin and glucose metabolism	Not reported
2017	Raed et al. [15]	70 (U.S.)	18 weeks	BP	Not reported
2017	McMullan et al. [16]	84 (U.S.)	Eight weeks	Not reported	BP
2018	Karefykalis et al. [26]	40 (Sweden)	26 weeks	Not reported	Dyslipidemia, insulin and glucose metabolism, abdominal obesity, prothrombotic state
2019	Angellotti et al. [24]	127 (U.S.)	48 weeks	Not reported	Dyslipidemia and Pro-inflammatory state

Limitation

Our systematic review of 14 research works has limitations. The most notable limitation is that the studies focused on different components of MetS, making it difficult to carry out like-for-like comparisons. For example, Khoja et al. used HbA1C to measure insulin and glucose metabolism, whereas the study by Mirhoseini et al. used FBS. Similarly, Raed et al. used carotid-femoral PWV and carotid-radial PWV to measure the BP, while McMullan et al. used PRA, Ag II, and 24-hour BP as indicators. As a result of these differences, we could only compare these studies on a higher level. Another limitation is that we do not have access to the detailed analyses and datasets used in these studies; therefore, we could not verify and critique the analytic approaches, nor could we perform a statistical meta-analysis. Last but not least, individual studies have their limitations, such as small sample size and insufficient study length.

## Conclusions

In our systematic review, we aimed to verify the overall relationship between Vit D and MetS, the associations between Vit D and each of the six major components of MetS, and the effects of Vit D supplementation on MetS components. Our review of three observational studies, one systematic review, and one analytical study verified the significant overall relationship between Vit D and MetS. Four out of five observational studies established significant associations between Vit D level and four main components of MetS, namely dyslipidemia, insulin, glucose metabolism, obesity and BMI, and BP. In addition, four out of seven RCT studies revealed significant effects of Vit D on BP, abdominal obesity, and insulin and glucose metabolism, which are the three core components of MetS. Although proinflammatory state and prothrombotic state are important components of MetS, the studies reviewed did not discover any significant association between Vit D level and each of these two components, nor did the studies establish any significant effects of Vit D supplementation on them.

Two of the RCT studies, namely the ones conducted by Cefalo et al. and Karefykalis et al., studied BMI and body fat content as the focal MetS components concerning Vit D level. These studies recruited relatively few participants (N=18 and N=40) and could be statistically underpowered in their findings. More RCT studies that explore the relationship between Vit D and obesity, as measured by BMI and body fat content, are needed. In addition, there is a need for future studies that perform subgroup analyses to account for the differences in sex, age group, the underlying VDD prevalence of each population; specific medical conditions of the participants such as pre-existing renal or liver issues that could affect their Vit D levels and MetS diagnoses; and other factors such as Vit D in daily dietary intake, passive vs. active lifestyle, and current medications.
